# The Effects of Meteorological Conditions on the Circadian Rhythm of Births

**DOI:** 10.1002/ajhb.70120

**Published:** 2025-08-07

**Authors:** Alessio Fornasin, Laura Rizzi, Giovanni Fonseca

**Affiliations:** ^1^ University of Udine Udine Italy

**Keywords:** 19th century, births, circadian rhythm, North‐Eastern Italy, weather

## Abstract

**Objective:**

The aim of this study is to evaluate the influences exerted by temperature on the circadian rhythm of births.

**Methods:**

In the past births occurred mainly between midnight and dawn, while today births tend to be less frequent during the night hours. Today, almost all births are hospitalized and, therefore, they may adapt to the organizational requirements of the health care structures and staff. This piece of research regards births in Udine, a city in north‐eastern Italy, recorded at the beginning of the 19th century. The data on births come from the French civil register. Weather data come from very detailed daily collection with three measurements per day. From the statistical point of view, we apply methods developed for circular data. In order to highlight relationships between time of birth and explanatory variables, we estimate MANOVA (multivariate analysis of variance) models and perform a statistical test for comparison between groups.

**Results:**

The test against the homogeneity of the hour‐of‐birth distributions across the 4 seasons is significant (*p* < 0.01). One‐to‐one comparison of the distributions is performed via Watson's two‐sample test on data for each couple of seasons: the Summer‐Autumn comparison is the only non‐significant test of homogeneity. Moreover, we consider daily temperatures in the bivariate model in several different ways and transformations. The daily temperature effect is more significant if considered as the difference between the evening temperature of the day before the birth and the mean daily temperature of the same day, over the nine‐year period 1807–1815. Finally, based on this division of the births, Watson's two‐sample homogeneity test for the distribution of the hour of birth is significant (*p* < 0.05).

**Conclusions:**

We found that the circadian rhythm of births is influenced by temperature, with an anticipation of the time of birth on warmer days. To our knowledge, our results are the first evidence of the effects of daily temperature on the time of birth.

## 1 Introduction

Childbirths are not uniformly distributed over 24 h. Today, births are more frequent during daylight hours, while in the past, they frequently took place at night. This change occurred during the last century, due to the increasing hospitalization of childbirths and due to fertility decline. Childbirth medicalization, typical of almost all births today, has led to a general change in the natural rhythms of births. Currently, women give birth to children mainly during working hours; then these events become less frequent during weekends and at night.

In European countries, the hospitalization of childbirths has become increasingly widespread over the last century and dominant in the period since World War 2. The patterns of this change have been heterogeneous across countries and territories, occurring mainly in urban areas that served as maternity care hubs for both city and surrounding rural populations. Hospitalization has led to the adoption of procedures devoted to reducing potentially risky situations for mother and child that usually anticipate and, more rarely, postpone the birth by a few days or even just a few hours.

Sometime, however, invasive practices, such as caesarean sections, are undertaken to define the moment of birth even in the absence of real necessity (absence of risk factors). This process has greatly reduced, or even eliminated, the opportunity for collecting information on the natural 24‐h birth cycle, given the limited observations on non‐induced childbirths.

The first studies defining daily birth cycle date back to the 19th century. However, this topic is still of interest for researchers given that its disruption can cause health consequences for mother and child. On these grounds, the present research aims to deepen our understanding of factors affecting the circadian rhythm of births and, in particular, the elements which trigger the causal sequence of events during childbirth.

The purpose of this study is to evaluate if and how the childbirth hour is affected by meteorological factors. To this end, it is necessary both to consider past births, when they took place outside clinics and hospitals, and to consider births with information on the precise time and with meteorological conditions recorded at least daily. These requirements were not easily available for the pre‐transitional demographic regime. However, this information is available for the city of Udine, located in northeastern Italy, in the period 1807–1815. This research is based on individual data from French civil status registers for births, while data on temperature and atmospheric pressure are taken from a very detailed set of information collected by the meteorologist Girolamo Venerio. The study is structured in five sections. Literature review is presented in the next section, while the third part is devoted to the description of the social, demographic, and environmental aspects of the city of Udine, of the sources of data, and of methodology. The fourth section reports the results, and the fifth one is devoted to discussion and our conclusions.

## 2 Materials and Methods

### 2.1 What Do We Know About the Circadian Rhythm of Births?

There is a substantial literature on the seasonal, weekly, and daily cyclicality of demographic phenomena. Today, the cyclicality of births depends mainly on social and economic factors. Couples may plan conception and thus, with some approximation, the date of birth, but births may also be partially conditioned by the organization of work in natal wards.

Thus today, births are, at least partially, planned in three main steps. Births' seasonality depends on couples' behavior, weekly cycles depend on health and hospital organization, and daily cyclicality is linked to the decrease of births above the first order. In the past, only seasonal cyclicality was linked to the social and economic aspects, such as the distribution of marriages during the year, then of first‐born conceptions, or the seasonality of labor, which was also correlated to weddings.

In a context where the management of childbirth was usually assigned to midwives without any medical training, the daily/weekly cyclicality and also any peculiar distribution of births during the days and weeks were not recorded. Since the first half of the 19th century, several scholars have noted that births were not evenly distributed throughout the day, but were usually concentrated in the first few hours after midnight. The idea, now accepted, that a greater concentration of births in this part of the day reflected the ‘natura’ course of the phenomenon started to be considered. The natural distribution of births during the day is attributed to biological factors that are the result of adaptation and selection processes. Uterus activity during labour is synchronized with the cycles of light and darkness, both through melatonin, which is secreted in the absence of light, and through other related hormones (Sharkey et al. 2009; Olcese et al. 2013; Chaney et al. 2018; McCarthy et al. 2019).

This non‐random distribution is related to past periods when delivering at a time when assistance was available was advantageous. Delivering during the afternoon exposed a mother to the risk of being away from home and other adults while she foraged alone for food (Jolly 1972; Trevathan 2011).

The first researcher who dealt with the circadian rhythm of births was Adolphe Quetelet (1827), who published the first series of data on the hour of childbirths. He observed the daily hours of births at the Saint‐Pierre Hospital in Brussels from 1811 to 1822. Quetelet found that more births occurred in the six hours after midnight than during other times of the day. In a later study, the Belgian statistician observed similar birth trends at both the Hospital de la Maternité in Paris and in a series of data related to the city of Hamburg (Quetelet 1835). Subsequently, other researchers published data on the hourly distribution of births in different European localities, almost all showing very similar patterns. Among the 890 births observed by the surgeon Walter Ranken in Airdrie, Scotland, the majority were recorded in the early hours of the morning (Ranken 1827). The distribution of births in Germany, in the city of Schmalkalden, between 1845 and 1848, showed a similar trend, with a maximum spike in the early hours of the morning and the lowest levels between noon and 2 pm (Danz and Fuchs 1848). All the data were collected by medical personnel and thus concerned medically assisted births. However, similar findings emerged from the research conducted on French civil status registers in the second half of the 19th century relating to a Paris *mairie* (Gauquelin 1959) and to the parish books of two localities in Bessarabia from 1828 to 1906 (Gietel‐Basten 2019).^1^

Many factors may affect the daily distribution of births. On the relationship between birth hour, season, and photoperiod, there are, however, a number of opinions. Discussions date back to Buek (1829, but we consider the data from Quetelet 1835) the first publication of the series of data on births distinct by season of occurrence. The result, with reference to the 1820s, does not show a clear seasonal pattern, although in winter there is a more pronounced concentration of births shortly after midnight.

More than a century later, when the hospitalization of childbirth became very common, some authors proposed two classifications of factors, environmental and social, that could determine the different daily distribution of births and labour in different seasons. Among the studies that have looked at this aspect there is Charles (1953), for the city of Birmingham in central England, and Colombo (1955) for the city of Padua, in northern Italy. These studies considered sunlight and temperature as environmental factors, and the routine imposed on mothers by work rhythms, mealtimes, and rest times. To evaluate the difference between the impacts of the social routine and of environment, scholars looked at periods close to the equinoxes (equal length of day and night) and solstices (maximum difference in duration). This allowed for an assessment of the length of daytime and the environmental temperature. The results have been compared with those relative to the routine of certain forms of social behavior that may be constant (the timing of meals) or variable (the rhythms of rest). These aspects have also been studied with regards to the introduction of solar time, which affects the social, but not the environmental factors (Charles 1953). However, no persuasive results emerged. In fact, Charles did not detect any seasonal differences in the hourly distribution of births and no evidence of photoperiod influence. Colombo suggested that ‘the differences in the total number of births at different times of the day in a specialized hospital should not be considered as indicative of a biological cycle. They depend, in fact, substantially on the ‘quality’ of the births, the parity, and the normal or pathological course, and, moreover, on the habits and organization of society’. Colombo concluded, therefore, by stating that ‘explanations from biological determinants and influences of the external physical environment of the hourly rhythm of onset of contractions seem to lose force and acquire those that refer to the rhythm of everyday life’.

More recent studies have pointed out different results. Varea and Fernández‐Cerezo (2014), using data from the Casa de Maternidad in Madrid from 1887 to 1892, assess that in a context without modern obstetric intervention and no artificial lighting, the time of birth depends on the length of the day. Then, in the two months close to the summer solstice, births resulted more frequently from 8 a.m. to 12 noon, and in the winter solstice from 4 to 8 a.m. According to the authors, light conditions influenced the time of birth. It was, however, unclear why winter births occurred in opposite light conditions to summer births.

One aspect in which the results of most studies agree is the relationship between time of birth and birth rank. To our knowledge, the first scholar who studied this relationship was Berlinski (1834), whose data were afterwards considered by Casper (1846). In a survey of 809 births that took place in the maternity home (*Gebäranstalt*) of the University of Berlin from 1830 to 1833, Berlinski, after noting that births occurred most frequently in the early hours of the morning, observed that the births of primiparous mothers followed the established pattern. Those of multiparous mothers, on the other hand, were more likely to take place between 6.00 p.m. and midnight^2^. Subsequent studies have contradicted these results and suggested the opposite, namely that primiparous women give birth more often in the afternoon, while multiparous women are more likely to give birth in the early morning (Charles 1953; Colombo 1955; Gauquelin 1959; Anderka et al. 2000; Mancuso et al. 2004). As noted below, these differences are largely due to the duration of labour. However, this different timing implies, as pointed out by demographers, that the hourly distribution of births varies according to fertility. As fertility increases, the frequency of births at night becomes greater (Colombo 1955).

With the increase in the hospitalization of deliveries, the collection of information on the start and duration of labour has been systematically implemented. This information plays a relevant role for the present research, as the start of labour marks the beginning of a chain of events (first contractions, painful contractions, breaking of the waters, etc.) that leads to birth. Before birth medicalization, the collection of this information on the start and duration of labour was very rare. In this context, too, the primacy belongs to Berlinski (1834), who observed that ‘Night has more power than day in procuring labour’, and that there was no particular variability in the time elapsed between the onset of labour and birth: labour lasted on average 20 h, if onset of labour took place during the day, and 18 if it took place at night.

Determining the onset of labour is much more difficult than establishing the time of delivery, and scholars have proposed different criteria here. Charles, in his study (1953) sets the onset of labour at the first painful contraction of the uterus followed by other contractions at regular intervals. In Colombo (1955), the start of labour is set at the first contractions, even in the case of the premature rupture of membranes. In Smolenski et al. (1972) it occurs with the ‘spontaneous onset of painful contractions and/or rupture of the foetal membranes’. The duration of labour depends, of course, on the time of its onset. According to the criterion adopted by Smolenski, the average duration is nine hours. But it changes in relation to the rank of the birth and, as pointed out before, primiparous women have shorter labour, the difference being in the order of three to five hours (Tilden et al. 2022). Some authors have wondered whether the duration of labour can be related to the time of its onset. Kaiser and Halberg (1962) found an inverse relationship between the time of the onset of labour and duration, i.e., that labour that starts late in the day leads to a shorter time to delivery, which would be the reason why births are concentrated in the early hours of the morning. In Cagnacci et al. (1998), on the other hand, the duration of labour did not appear to depend on the time of onset, although the time of delivery did depend on the duration of labour. The onset of labour also follows a circadian rhythm, in which, as a rule, the acrophase is most frequently between midnight and 2 a.m. (Heres et al. 2000; Cooperstock et al. 1987; Jolly 1972). This rhythm depends on the sequence of events in labour itself, i.e., whether it begins with contractions or with the breaking of the waters (Moskon et al. 2024). The duration of labour, moreover, depends on the specific conditions of the pregnancy and birth; the duration is different if the birth is premature, if the baby is stillborn, if there are complications during the last stages of pregnancy, if the mother is subjected to particular stress conditions, etc. (Heres et al. 2000).

Many studies in the last years have continued to examine the changes occurring in the circadian cycle of birth and labor. As we have implied several times, most of the authors who have dealt with it have stressed the role of the increasing hospitalization of childbirth, which shot up after the Second World War. Hospitalization started in urban areas and then spread to the countryside and, finally, became almost universal. One of the consequences of hospitalization has been the introduction of induced labor and cesarean sections, resulting in a revolution in the timing of birth. Since the 1950s, numerous studies have focused on this new trend. Charles (1953) for Birmingham and Colombo (1955) for Padua, studying the differences between home and hospital births, noted that at home they were more frequent in the early morning hours, while in hospital they occurred more often in the early afternoon. Peter King (1956), considering data from five hospitals in different cities in the United States, reported that where labor was induced the daily distribution of births underwent a marked change, being arranged between the late morning and afternoon hours. In France, the statistical analysis of data from a large Parisian hospital pointed to the shift from a concentration of births in the early morning hours to a more uniform distribution in the 1950s (Gauquelin 1959).

Numerous other studies have confirmed this trend in many other countries, at least with reference to low‐risk births, emphasizing how the 24‐h rhythms reflect the daily activity of hospital personnel (Glattre and Bjerkedal 1983; Macfarlane et al. 2019; Varea et al. 2016; Gietel‐Basten 2019; Anderka et al. 2000). Finally, as we noted above, there is another factor that affects this new cyclicity, namely fertility decline with the consequent dominance of first‐born births (Varea et al. 2016).

Since almost all births today are hospitalized, it is difficult with current data to isolate external'natura' factors that affect the timing of births. There are few studies in this area. Some, for example, were concerned with the relationships between temperature and atmospheric precipitation and the seasonality of births (Lam and Miron 1996; Manfredini 2009). Others investigated the possible connections between meteorological factors and the incidence of premature births (Yackerson et al. 2008). Research dealing with the relationships between meteorological aspects and the circadian rhythm of births and labor is even rarer, and the results are rather uncertain. Driscoll and Merker (1984), for example, identified some weak evidence that human parturition is influenced by meteorological variables.

This relationship is evident in the presence of cold fronts, i.e., when there is a substantial reduction in temperature from one day to the next, with strong winds and low atmospheric pressure. In a later study by Driscoll (1995), however, applying the same methodology on another dataset, no confirmation of this relationship was found. Noller et al. (1996), investigating changes in atmospheric pressure, found a correlation between a decrease in pressure and the onset of labour. The relationship is significant but negligible. Hirsch et al. (2011), using data on temperature, pressure and humidity, noted that ‘Meteorological factors are associated with the timing of parturition, but the magnitude of this association is small’.

If we want to recap how the cyclical nature of births has evolved over the last two centuries, there has been a shift from the'natura' rhythms of childbirth to'artificia' rhythms. In the 19th century, the rhythm was natural; in the 20th, artificial. With regard to the empirical evidence available for the 19th century, however, besides the two already mentioned studies by Gauquelin (1959) and Gietel‐Basten (2019), all the others use data on births that took place in a hospital. Thus, we cannot verify that they took place under'natura' conditions.

In Berlinski's work (1834), for example, the time series of births in the Berlin hospital is published with a distinction between natural births and ‘*ope artis finiti*’ births, an expression indicating those that occurred under medical care and intervention. The latter are more than 15% of the total. This distinction, not made in the other works from the 19th century, begs several questions on the representativeness of the births considered and on possible selection factors. Who were the women who gave birth in the hospital and for what reason were they admitted? The Berlin data suggests that among these women an above‐average number carried a high‐risk pregnancy. The same proposition was made by Colombo (1955), who, in addition, suggested that among the women who gave birth in the Obstetrical‐Gynecological Clinic of the University of Padua, 40% were primiparous, and that most came from the city, hence from the neighborhoods around the hospital. The place of residence, therefore, could also be a selection factor. Women who lived in the city could reach the clinic much more easily than those from the countryside, and there were also more urgent admissions among the former than among the latter.

If we point out these selection effects for the nineteenth century, moving into the twentieth century, the problem becomes even more striking. More and more women are hospitalized and, moreover, patients are kept in controlled and relatively homogeneous environmental conditions, starting with room temperature and artificial lighting. In addition, as we have seen, there are an increasing number of women who have undergone medical interventions that have altered the natural course of labor and the timing of delivery. If we want to consider labor and childbirth in their spontaneous form, we must consider them outside hospital facilities. It is therefore necessary to investigate the past rather than the present.

### 2.2 Setting and Sources

#### 2.2.1 Udine in the Early 19th Century

**FIGURE 1:**
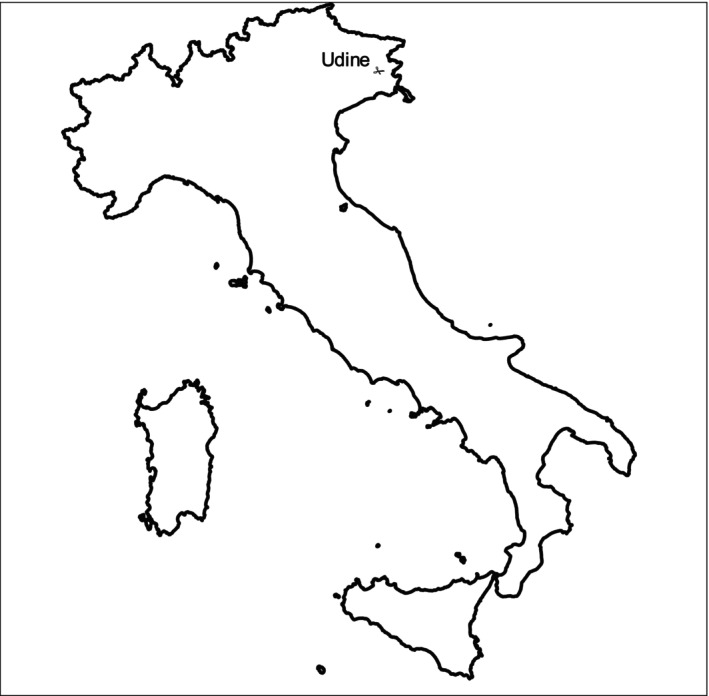
Udine in the Italian context.

Udine is a small city in the North‐East of Italy which, in the early 19th century, had around 13 000 inhabitants and faced a crude birth rate of about 36.4 per thousand and a crude death rate of 35.2 per thousand. It was the only urban centre in Friuli, the region in the eastern part of the Napoleonic Kingdom of Italy and, until its fall in 1797, it had been part of the *Stato di Terraferma* of the Republic of Venice (Figure 1).

Despite its low population, Udine was, in socioeconomic terms, composite. Numerous artisan and commercial activities coexisted within its walls, which served a large rural hinterland. There were many mills along the two main canals that ran through the city, and there were iron workshops and tanneries. Textiles were important, and there were many resident spinners and weavers. Udine was also an important administrative center. The observed period faced an increase in central state bureaucracy, and many white‐collar workers migrated to Udine from other Italian territories. The most important families and landowners of Friuli lived within the city walls, but there were also modest farming families who cultivated land whose activities ranged from modest intra‐mural market gardening to more extensive farming. From the geographical point of view, Udine was located in the center of a large plain bounded to the north by the Alps and to the south by the Adriatic Sea. Udine lies in an area with a temperate continental climate, with fairly high temperatures in summer, and it becomes relatively cold in winter (Gentilli 1964).

#### 2.2.2 Demographic Data: The Napoleonic Civil Registers

The availability and quality of data on Udine for the first two decades of the 19th century, fundamental for analyzing the relationship between births and meteorological factors, is exceptional. The birth of the Kingdom of Italy in 1806 saw the introduction of the Napoleonic Civil Code throughout the country, which led to civil registers for births, deaths, and marriages. The present study uses the birth registers, which were kept from January 1st, 1807, to December 31st, 1815. These records report for each birth the date of the event, the name and surname of each newborn, and socioeconomic information, such as the occupation of the parents. Recordings do not include parity of births. Hence, unfortunately, we are not able to consider this factor in the analysis, but let us assume that approximately 80% (or more) of the registrations refer to second‐born children and above^3^.

**FIGURE 2:**
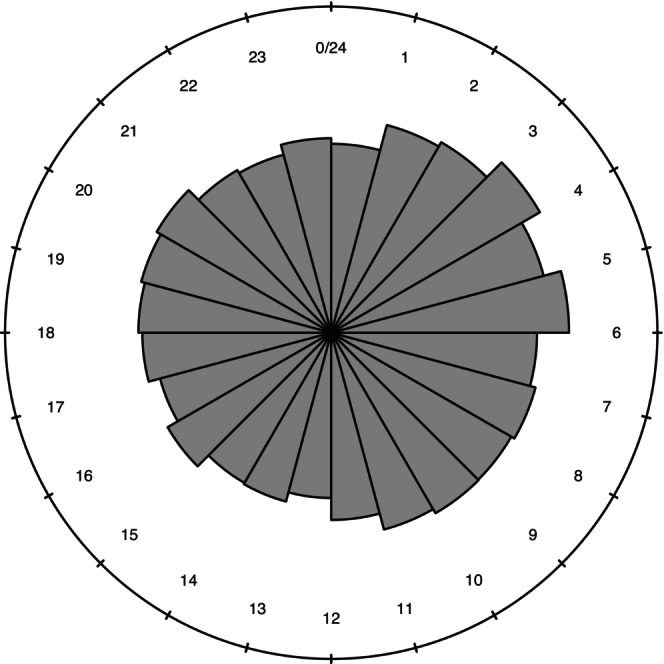
Hourly distribution of births in Udine (1807–1815).

Records report the house number, which allows us to localize births on the territory and exclude from the analysis the very few events in the hospital^4^. Given the aims of our study, of particular interest is the indication of the hour of birth, which began to be collected regularly in the French civil status registers. Therefore, this detailed information represents a source of absolute relevance for the study of demographic phenomena on a sub‐daily basis. Another very important aspect is related to the measurement of time, which became uniform across a large part of the continent with the expansion of Napoleonic influence in Europe. From this moment onwards, the ‘French‐styl’ computation of hours was adopted throughout Italy, with the division of the day into 12 midday and 12 afternoon hours (Dominici and Marcelli 1979). Not all the available information has been taken into account. In fact, we consider only newborns whose place of birth is identifiable in the municipal territory. We need also to stress that, as in all Italian cities at the time, Udine had a hospital where foundlings were left. The number of children abandoned in this period stands at 1477, or about a quarter of all births registered. These foundlings are excluded from the analysis because their date and hour of birth are unknown. However, we must also consider that the vast majority of them, about 4%, were born in rural villages rather than in Udine (Fornasin and Rizzi 2022). Moreover, a relevant aspect of the hourly collection of births is the bias in recording the midnight events, due to the ambiguity on the time of transition from one day to the next (Gauquelin 1959; Gietel‐Basten 2019; Kaiser and Halberg 1962). The quality of collected data is affected also by factors linked to beliefs and superstitions. In a study of Somogyi (1953), based on almost 900,000 births thatappened in Italy in 1951, a concentration of events in the first hour of the day, in the round number hours and, more generally, in those ending with an even number was observed. While midnight and 5 p.m., denoted as 17:00 in the Italian style, were evidently considered unlucky numbers and are poorly represented. The distribution of events resulted less skewed relative to births in hospitals or with medical assistance. Figure 2 reports the hourly distribution of the births' series considered in this research. Even if more details are described below, a higher incidence of childbirth in the early hours of the morning, particularly between 4 and 6 a.m., is already observable. Comparing the distribution of events in our series with others that are less informative, a few data points can be set out: the first hour of the day is not inflated to the detriment of the last one; the preference towards even hours is mainly found at 4 and 6 o'clock and, similarly, the odd hours do not show very deep reductions; finally, even births at 5 p.m. (an unlucky hour), although fewer in number than those at the previous and following hours, do not show a marked under‐recording.

#### 2.2.3 Weather Recordings: The Data Collection of Girolamo Venerio

Meteorological data sources for Udine in the period are particularly rich. This is due to the exceptional data collection of the meteorologist Girolamo Venerio. Venerio collected data on atmospheric pressure, temperature, rainfall, and wind direction at different hours of each day. He collected this information over more than forty years, starting on January 1st, 1802. This series stops the day Girolamo Venerio died, on March 4th, 1843. His measurements are very accurate. Over the 40 years, he carried out daily checks on the functionality of the instruments and on their calibration. He also made an effort to be consistent both in terms of the reading times and places. Every modification was documented by Venerio in order to evaluate and quantify the effects on the quality and comparability of the data (Cittadella 2016).

**FIGURE 3:**
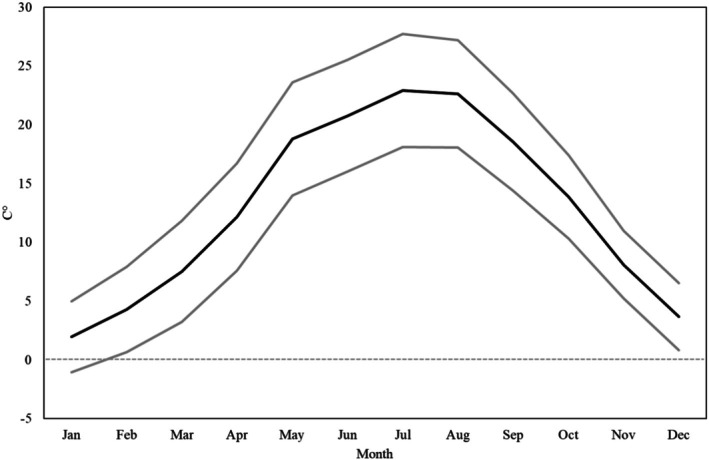
Monthly temperatures (average, minimum and maximum) in Udine in degrees Celsius (1807–1815).

During the first two decades of the 1800s, weather data were collected by Venerio three times each day (at dawn, at noon and at sunset). The temperature was measured at sunrise, between 1 and 3 p.m., and at 10 p.m., by making available the measurements at times of the day corresponding to the minimum (sunrise) and maximum (after midday) level of temperature. A similar schedule was adopted for the atmospheric pressure, which in the afternoon was measured between 14:30 and 16:00. The average, minimum, and maximum monthly temperatures, relative to Venerio's entries for the years considered in the study, are reported in Figure 3.

### 2.3 Methods

In order to highlight the relationship between the time of birth and factors influencing circadian rhythm, we apply methods developed for circular data. This setting is useful when the support of the variable of interest is periodic, i.e., the extremes of the support coincide, and, hence, data can be displayed on a circle instead of as an interval of the real line. In our case, the support is the interval [0,24] representing the day time, and time 0 and 24 are the extremes which overlap between two consecutive days. Examples of such types of variables are daily times, months of the year, directions, and so on. In these cases, data are expressed as points on a circle and hence can be identified by the value of an angle^5^.

Special care is needed when performing statistical analysis on this kind of data, and the usual methods may not be appropriate. Consider, for instance, the case of computing the sample mean of the following two times of birth: 23.55 and 0.05. The correct solution is midnight, but the usual rule of computation of the mean gives noon.

The response variable, time of birth, was observed as a binned continuous variable with 24 values (see Figure 2). We assume in the following that all the births in an hour were labeled by the later hour, e.g., all the births between 3 and 4 were labeled as 4 in the dataset. When needed by the assumptions of tests and model estimation, these are performed both on the original observed variable and on the simulated observed continuous variable, obtained by uniformly assigning the counts of births at each hour to the corresponding one‐hour time interval. No discrepancy is observed in the results between the two sets of observations.

In order to highlight relationships between time of birth and explanatory variables, we estimate MANOVA (multivariate analysis of variance) models and perform a statistical test for comparison between groups. The MANOVA models adopt, as the response variable, the bivariate vector of observations obtained as the sine‐cosine value of the angle representing each time observation.^6^

To assess the role of external conditions on the time of birth, we adopt an'experimental approac'. There is no theoretical frame of reference in the literature, as the relationships between'natura' exogenous factors and time of birth have not yet been checked. Of course, our methodological approach is limited by data availability. In historical context analyses, it is generally difficult to get the information necessary for estimating the models adopted in our research. This study represents an exception since the detail and quality of the series employed are very similar to data collected in much more recent times. Our dataset concerns 4455 records of the time of birth: all events occurred in dwellings within the city walls of Udine between 1807 and 1815. The first explanatory factor considered in the model is the season of the year of each birth; the relatively low number of data did not allow us to consider shorter periods of time. However, two models were performed with different approaches to data organization. One criterion, aimed at investigating the role of meteorological variables, was based on clustering of data according to climatic seasons. Another, aimed at assessing the relationship with the photoperiod, was constructed by evaluating the periods of the year according to luminosity. The second explanatory variable included is based on temperature measurements.

The short‐term relationship between temperature and time of birth has not been, to our knowledge, thoroughly investigated yet. To accurately assess this relationship, we needed to separate daily temperature variations from broader seasonal patterns. We therefore manipulated the data to neutralize seasonal effects. For this reason, we considered in the analysis the difference between the temperature measured on each day D and the average of the temperatures collected on the same day of all the nine years observed. Therefore, a positive value of such variables on a specific day D denotes a higher temperature than the average one on all day D in the years 1807–1815, while a negative value points to a lower temperature. The same criterion has been adopted for temperatures measured at the three separate times of the day and for the daily average. The third variable is atmospheric pressure, which represents the meteorological aspect that most often has been taken into account in the literature to evaluate the relationships with the time of birth and, above all, the start of labour. In this case, data have been considered without further manipulation, as at low altitudes, pressure changes very little in relation to temperature, which is the aspect that differentiates Udine throughout the year.

## 3 Results

In detail, following the circular data approach, we define a bivariate linear regression model where response variables *Y* ^1^ and *Y* ^2^ are, respectively, the cosin and sin transformation of the time of birth expressed as radian angle. Explanatory variables are the season, as a factor with 4 levels (included in the model with the 3 dummy variables S_2_, S_3_ and S_4_ relative to spring, summer and autumn season, respectively), and the continuous covariates.

**FIGURE 4:**
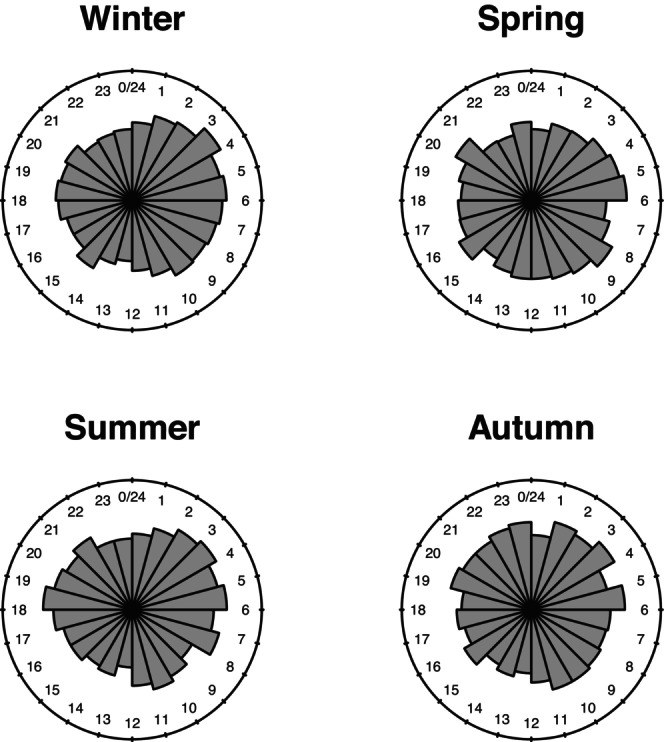
Hourly distribution of births in Udine across the four seasons (1807–1815).

We began investigating the effect of seasons. Rose diagrams of time of birth across the four seasons appear quite different, indicating the presence of an effect of the season on the distribution of the time of birth (Figure 4).

We adopted the MANOVA approach to compare the four seasonal distributions of the time of birth. The two equations defining the systematic component of the model are:

$$Y_{i}^{1}=\alpha^{1}+\beta_{2}^{1}S_{2,i}+\beta_{3}^{1}S_{3,i}+\beta_{4}^{1}S_{4,i}$$

$$Y_{i}^{2}=\alpha^{2}+\beta_{2}^{2}S_{2,i}+\beta_{3}^{2}S_{3,i}+\beta_{4}^{2}S_{4,i}$$

The test against the homogeneity of the distributions among the 4 groups is significant (*p* < 0.01), hence a seasonal effect appears to be present, although the fitting of the estimated model is small. Then, in order to compare one‐to‐one the distributions of the response, Watson's two sample test was performed on data for each couple of seasons. Only the Summer–Autumn comparison reveals a non‐significant homogeneity. In conclusion, the distribution of the time of birth is associated with the season of the year. Another factor whose effect is connected with seasonality is the photoperiod. To measure this effect, we considered a factor with two levels for the groups of months of birth November–April and May–October. Watson's two sample homogeneity test is again significant (*p* < 0.05). As a final remark, when both the season of birth and photoperiod are added in the MANOVA model, the effect of seasonality collects almost all the effect of the photoperiod factor, whose contribution, interaction included, is no longer significant.

Moreover, in order to evaluate the effect of both seasonality and photoperiod, we considered the mean time of birth (in radians) for each group. The results are reported in Tables 1 and 2.

**TABLE 1:** Average time of birth in radians and hours (meteorological seasons).

Season	Radians	Hours
Winter	1.38	5.30
Spring	1.85	7.08
Summer	0.97	3.72
Autumn	0.85	3.27

**TABLE 2:** Average time of birth in radians and hours (photoperiod).

Photoperiod	Radians	Hours
November–April	1.42	5.44
May–October	1.00	3.84

A difference of 0.1 rad corresponds to almost 23 min in the time scale and, hence, as an example, we can see that, on average, the time of birth is anticipated by about one and a half hours during the summer with respect to the winter season, and also for the months between May and October, with respect to births between November and April.

Finally, we investigated the dependence of the time of birth on weather aspects like daily temperatures and atmospheric pressure. We used again the MANOVA decomposition of deviance considering seasons, temperatures, pressure, and their interactions.

The atmospheric pressure has a non‐significant effect. We included daily temperatures in the model in several different ways and transformations. The daily temperature effects are more significant if considered as the difference between the evening temperature of the day before the birth and the mean daily temperature of the same day, over the nine‐year period 1807–1815 (denoted as the covariate *X* afterwards). We defined two different models considering, as weather explanatory variables, respectively, a continuous covariate *X* (in Model 1) or the transformation of *X* defined as a factor with two levels as *X* > 0 or *X* < 0 (in Model 2). Interaction effects do not result as being significant.

Hence, the system of two equations defining the systematic component of the final model is

$$Y_{i}^{1}=\alpha^{1}+\beta_{2}^{1}S_{2,i}+\beta_{3}^{1}S_{3,i}+\beta_{4}^{1}S_{4,i}+c^{1}X_{i}$$

$$Y_{i}^{2}=\alpha^{2}+\beta_{2}^{2}S_{2,i}+\beta_{3}^{2}S_{3,i}+\beta_{4}^{2}S_{4,i}+c^{2}X_{i}$$

As shown in Table 3, the results of the analysis show high significance (*p* < 0.005) of the seasons, as expected, and also the relevance of covariate X in both model specifications (*p* < 0.005). Again, as for the previous model, we note a low fitting level of the estimated model.

**TABLE 3:** MANOVA deviance decomposition.

Model 1	*p*	Model 2	*p*
Intercept	< 2.2e−16	Intercept	< 2.2e−16
Season	0.003579	Season	0.003572
X (covariate)	0.002161	X > 0 (factor)	0.001556

In the sequel, we highlight the effect of temperature on time of birth, splitting the observations of time of birth into two groups depending on the corresponding value of X, negative or positive. Watson's two‐sample homogeneity test is again significant (*p* < 0.05) in the comparison of the distribution of times of birth between these two groups. Moreover, we considered the average time of birth if the day before was colder or warmer than expected (Table 4). Births occurred about half an hour earlier on days following warmer weather.

**TABLE 4:** Average time of birth for X < 0 and X > 0.

	Radians	Hours
X < 0	1.31	5.03
X > 0	1.19	4.57
Difference (min)		27

Finally, even if the interaction components in Model 1 and 2 showed a non‐significant role, we evaluated the effect of temperature in the different seasons (Table 5). It is interesting to note that warmer days were associated with an anticipation of almost two hours in the average time for the next day's birth during the winter. But this anticipation results negligible during the summer.

**TABLE 5:** Effect of temperature in Winter and Summer.

	Winter		Summer	
	Radians	Hours	Radians	Hours
X < 0	1.64	6.28	0.99	3.79
X > 0	1.15	4.40	0.96	3.67
Difference (minutes)		112		7

## 4 Discussion and Conclusions

The results of this study, concur with the main literature, in confirming the existence of a natural circadian rhythm of births.

At the beginning of the 19th century, the relative majority of births in Udine occurred in the first hours of the day, from midnight to 6 AM. As pointed out in the literature review, one aspect of debate among researchers was around the differences in the circadian rhythm at different periods of the year. In our study on births in Udine, these differences have been assessed in the period considered. The'classi' distribution of daily birth hours is particularly marked in the winter and summer periods. Moreover, the analysis of the distributions of the hours of birth shows a weak association with the photoperiod, and the effect of the seasons on the hour of birth is to a greater extent affected by temperature. However, in terms of average time at birth, spring and autumn showed the most delayed and early births. The short‐term relationships between temperature or atmospheric pressure and the time of birth lead us to exclude, against the literature, the role of pressure. The delaying effect of temperature lower than average, and, on the contrary, a birth anticipation, if higher, has been assessed. Our model also showed a stronger effect of temperature when we considered the deviations from the historical average temperature of the day before birth. This link between the time of birth and the temperature of the day before suggests that the relationship was related to the onset of labour, rather than to the hour of birth. We note that the fitting of the estimated models for the MANOVA decomposition is small, possibly due to the high dispersion of the response observations.

The results of this study are believed to shed new light on the interactions between the external environment and biological mechanisms. At the moment, we are not able to determine how the different factors interact with each other. In the literature, the emphasis is placed on the fact that the circadian rhythm of labor, and therefore of births, is the result of the adaptation and selection of biological processes that are synchronized with the cycles of light and darkness. These processes, consequently, act on the time scale defined by the human presence on Earth, and therefore should be almost immutable. In our work, however, an absolutely relevant role is played by temperature. From this perspective, adaptation and selection processes must also be read in the short‐term perspective defined by the action of meteorological variables. But, at the same time, there are other questions. On the grounds of medical research, we should look more carefully at which biological mechanisms are triggered by temperatures. On the demographic level, it would be important to ascertain how these aspects might affect the survival of newborn babies.

Further insights would be relevant for the health effects for mother and child of the transformation of the nocturnal pattern of birth into a diurnal one. On the one hand, it might cause a reduction in the mother‐baby bond, thus affecting breastfeeding (Bernis and Varea 2012). This would have had consequences for women's and children's health. It might, similarly, contribute to the increase of preterm and low birth weight deliveries, reducing breastfeeding, and later healthy life reductions (Bernis and Varea 2012). The greater number of still‐births that were observed diurnally might indicate that there were problems associated with delivery at ‘unusual’ times (Minors and Waterhouse 1981).

## Ethics Statement

The authors have nothing to report.

## Conflicts of Interest

The authors declare no conflicts of interest.

## Data Availability

The data that support the findings of this study are available from the corresponding author upon reasonable request.

## Endnotes

^1^
Further examples of 19th‐century observations are included in Oesterlen (1874), Fasler‐Segi (1951); Colombo (1955); Longo and Yellon (1988).

^2^
We note that in the table reported by the author first‐born children outnumbered those of all other orders combined, which suggests that either *primiparae* gave birth much more often in hospitals, or that fertility at Schmalkalden was very low, or that the survey is affected by errors.

^3^
We assume that fertility in Udine was similar to that observed in the Italian context at the end of early modern period (Livi‐Bacci 1977). This assumption is consistent with the ratio of newborns with the same mother to newborns with unobserved siblings in our source, given that the observation period spans only ten years.

^4^
We also note that the civil register records the proper time of birth of a newborn instead of the time of its baptism, as happen in the parish registers.

^5^
For a comprehensive exposition on the subject, see, among others, Pewsey et al. (2013), Ley and Verdebout (2017) and Jammalamadaka and SenGupta (2001).

^6^
For a comprehensive exposition on tests for comparison and MANOVA approach, see Landler et al. (2021).
